# Garbage codes in the Norwegian Cause of Death Registry 1996–2019

**DOI:** 10.1186/s12889-022-13693-w

**Published:** 2022-07-07

**Authors:** Christian Lycke Ellingsen, G. Cecilie Alfsen, Marta Ebbing, Anne Gro Pedersen, Gerhard Sulo, Stein Emil Vollset, Geir Sverre Braut

**Affiliations:** 1 Department of Pathology, Stavanger University Hospital, PO Box 8100, N-4068 Stavanger, Norway; 2 Department of Global Public Health and Primary Care, University of Bergen, PO Box 7804, N-5020 Bergen, Norway; 3 Department of Pathology, Akershus University Hospital, PO Box 1000, N-1478 Lørenskog, Norway; 4 Faculty of Medicine, University of Oslo, PO Box 1078, Blindern, N-0316 Oslo, Norway; 5 Department of Research and Development, Haukeland University Hospital, PO Box 1400, N-5021 Bergen, Norway; 6 Department for Health Data and Collection, Norwegian Institute of Public Health, PO Box 973, Sentrum, N-5808 Bergen, Norway; 7 Centre for Disease Burden, Norwegian Institute of Public Health, PO Box 973, Sentrum, N-5808 Bergen, Norway; 8 Department of Health Metrics Sciences and Institute for Health Metrics and Evaluation, University of Washington, 3980 15th Ave NE, Seattle, WA 98195 USA; 9 Department of Research, Stavanger University Hospital, PO Box 8100, N-4068 Stavanger, Norway

**Keywords:** Cause of death, Death certificate, Cause of death register, Garbage code, Non-informative code

## Abstract

**Background:**

Reliable statistics on the underlying cause of death are essential for monitoring the health in a population. When there is insufficient information to identify the true underlying cause of death, the death will be classified using less informative codes, garbage codes. If many deaths are assigned a garbage code, the information value of the cause-of-death statistics is reduced. The aim of this study was to analyse the use of garbage codes in the Norwegian Cause of Death Registry (NCoDR).

**Methods:**

Data from NCoDR on all deaths among Norwegian residents in the years 1996–2019 were used to describe the occurrence of garbage codes. We used logistic regression analyses to identify determinants for the use of garbage codes. Possible explanatory factors were year of death, sex, age of death, place of death and whether an autopsy was performed.

**Results:**

A total of 29.0% (290,469/1,000,128) of the deaths were coded with a garbage code; 14.1% (140,804/1,000,128) with a major and 15.0% (149,665/1,000,128) with a minor garbage code. The five most common major garbage codes overall were ICD-10 codes I50 (heart failure), R96 (sudden death), R54 (senility), X59 (exposure to unspecified factor), and A41 (other sepsis). The most prevalent minor garbage codes were I64 (unspecified stroke), J18 (unspecified pneumonia), C80 (malignant neoplasm with unknown primary site), E14 (unspecified diabetes mellitus), and I69 (sequelae of cerebrovascular disease).

The most important determinants for the use of garbage codes were the age of the deceased (OR 17.4 for age ≥ 90 vs age < 1) and death outside hospital (OR 2.08 for unknown place of death vs hospital).

**Conclusion:**

Over a 24-year period, garbage codes were used in 29.0% of all deaths. The most important determinants of a death to be assigned a garbage code were advanced age and place of death outside hospital. Knowledge of the national epidemiological situation, as well as the rules and guidelines for mortality coding, is essential for understanding the prevalence and distribution of garbage codes, in order to rely on vital statistics.

**Supplementary Information:**

The online version contains supplementary material available at 10.1186/s12889-022-13693-w.

## Background

Reliable vital statistics on the numbers of births and deaths – including causes of death – are essential for monitoring the health in a population [1], but not all cause of death data are fit for purpose [2]. The World Health Organization (WHO) defines the underlying cause of death as: “(a) the disease or injury which initiated the train of morbid events leading directly to death, or (b) the circumstances of the accident or violence which produced the fatal injury” [3]. It is the underlying cause of death that gives most information on the aetiology and thus possible targets for prevention. The International Statistical Classification of Diseases and Related Health Problems, 10th Revision (ICD-10) [4], does not only provide entities suitable for stating the (underlying) cause of death, but also for non-fatal diseases, for symptoms and signs or for conditions that could be an intermediate or terminal complication.

When there is insufficient information on the death certificate to identify the true underlying cause of death, the death will be classified using less informative codes. In the instruction manual for ICD-10, there are lists of ill-defined conditions and conditions unlikely to cause death [3], and these should be avoided, if possible. The term “garbage codes” was introduced by Murray and Lopez in 1996 as part of the Global Burden of Disease (GBD) framework to describe codes that are not useful for public health analysis [5, 6]. If many deaths are assigned a garbage code as the underlying cause of death, the true mortality pattern may be biased. In studies assessing the quality of cause of death data, the proportion of deaths assigned an ill-defined or garbage code has been one of the parameters used.

The list of garbage codes has been developed during the iterations of the GBD analyses, reflecting changes in the view of the origin and public health relevance [5, 7, 8]. In the current definition of garbage codes according to the GBD, there are 4 levels of garbage codes, reflecting the severity of public health implications. For level 1, the true underlying cause of death might belong to any of the three broad groups of causes of death (communicable, maternal, neonatal and nutritional disease; non-communicable diseases; injuries), and the information value of the garbage code is thus very limited. For level 2, the true underlying cause of death might belong to one (or at most two) of the three broad groups of causes of death. For level 3, the true underlying cause of death is likely to be within the same ICD chapter, and for level 4 the true underlying cause of death is likely to be within a single disease or injury category [6, 8]. For level 3 and 4, the spectrum of possible true underlying cause of death is narrower, and the garbage code has at least some information value.

Level 1 and 2 are major garbage codes, while level 3 and 4 are minor garbage codes. Examples of major garbage codes are sudden death, heart failure and unspecified sepsis, and of minor garbage codes unspecified stroke and cancer of unknown primary site.

The quality of the data in the Norwegian Cause of Death Registry (NCoDR) has been ranked as “medium” to “high” [9–12]. In 1980–2017, between 8 and 16% of the cases in NCoDR has been assigned a major garbage code, with the highest proportions in the more recent years. The closest neighbouring countries, Denmark and Sweden, have similar figures. Finland, Hungary and New Zealand are among countries with lowest proportion, 4–6% major garbage codes [7] (The numbers can be found in the supplementary appendix to the referenced article.)

### Aim

Our aim was to provide an in depth study of garbage codes in Norwegian cause of death data from 1996 to 2019.

1. Investigate the magnitude and pattern of use of garbage codes in the Norwegian Cause of Death Registry.
2. In the deaths coded with a garbage code as the underlying cause of death, are there other, more informative diagnoses (“non-garbage codes”) elsewhere on the death certificate?

## Materials and methods

### Materials

We used data from the Norwegian Cause of Death Registry (NCoDR) [13], on all deaths among Norwegian residents in the years 1996–2019 (*N* = 1,013,802). We chose 1996, when ICD-10 was introduced in the registry, as the start of the study period. We used the following variables: calendar year of death, sex, age at death, underlying cause of death (ICD-10 code) as well as all diagnoses entered on the death certificate (ICD-10), the (type of) place of death, and whether an autopsy (forensic or medical) was performed. The NCoDR selects the underlying cause of death according to the rules and guidelines provided by the WHO (ICD-10) [3], using the IRIS software [14]. A brief description of the processing at NCoDR has been published earlier [13]. Until 2017, all deaths were certified manually, on paper. Electronic certification of death was gradually introduced with a pilot in 2017, in the beginning available to only some hospitals and municipalities. It was not compulsory until January 2022. In 2017, 1 death was electronically certified, 75 in 2018, and in 2019 (the last year of the study period), 1231, 3% of the deaths were electronically certified. (The proportion increased to 37% in 2020 and 79% in 2021 (the last year with a dual system) (AG Pedersen, NCoDR, personal communication).)

Data from both manual and electronic certification was used, but the dataset does not contain information on which deaths that were certified electronically or on paper.

From the Global Burden of Disease Study (GBD), we used the mapping list from ICD-10 codes to the GBD cause list, including the list of garbage codes [7] (Table S4 in the supplemental material).

### Methods

Garbage codes in GBD class 1 and 2 were defined as major garbage codes, class 3 and 4 as minor. For tabulation of non-garbage codes, we used level 3 of the GBD cause list. For descriptive purposes, we grouped garbage codes that only differed in the fourth character of the ICD-10 code. In cases where both garbage and non-garbage codes were defined within the same 3-digit ICD-10 level, only the garbage codes were counted.

We used logistic regression analyses to identify determinants for the use of garbage codes. The outcome variables were whether the death was assigned a garbage code (any garbage code, major or minor) as the underlying cause of death. Possible explanatory factors were calendar year of death in 5 groups (4 or 5 year), sex, age of death in 7 groups, the (type of) place of death in five groups (hospital, nursing home, at home, other known, unknown), and whether an autopsy (either medical or forensic) was performed.

We used direct age standardization with the distribution of age of death in Norway 2015 as the age standard.

For all statistical analyses, we used R (version 4.0.4) and RStudio (version 1.4.1103) with additional packages from epitools and the Tidyverse collection [15–17]. We used Wilson’s method for calculating confidence intervals for proportions. For logistic regression, we calculated odds ratios with 95% confidence interval, likelihood ratio statistics (−2LogLikelihood) and two-sided *p* values. A two-sided *p* value < 0.05 was considered statistically significant.

## Results

### Overview over the data material

During 1996–2019, NCoDR had registered 1,013,802 deaths in Norwegian residents. After removal of deaths with missing death certificates, 1,000,128 (98.7%) remained, 513,851 women (51.4%) and 486,277 men (48.6%). The number of deaths each year varied between 39,110 (2019) and 44,825 (1999). During the study period, the median age of death rose from 82 to 85 year in women, and from 76 to 79 years in men. 50% of the deaths (Q1-Q3) in women occurred in the age interval 76–90 years, in men 68–85 years. The proportion of deaths occurring in hospitals declined from 40.9% (1996) to 29.5% (2019), whereas the proportion occurring in nursing homes rose from 36.8 to 52.6%.

For the entire study period, 29.0% (290,469/1,000,128) of the deaths were coded with a garbage code; 140,804 (14.1%) with a major and 149,665 (15.0%) with a minor garbage code.

### The most common garbage codes

Table 1 shows the most used major and minor garbage codes. The three most common major garbage codes were I50 (heart failure), R96 (sudden death), and R54 (senility), together accounting for 43.4% of the major garbage codes. The most common minor garbage codes were I64 (unspecified stroke), J18 (unspecified pneumonia), and C80 (malignant neoplasm with unknown primary site), together 64.6% of minor garbage codes. We found no considerable sex differences in the overall ranking.

**Table 1:** The most common garbage codes in Norway 1996–2019

*Diagnostic code*	*N*	*Percent of all deaths (95% CI)*	*Percent of GC in group*
**ALL DEATHS,** ***N*** **= 1,000,128**			
**Major GC**	*140,804*	*14.1 (14.0–14.1)*	
I50 Heart failure	36,683	3.7 (3.6–3.7)	26.1
R96 Sudden death	14,127	1.4 (1.4–1.4)	10.0
R54 Senility	10,298	1.0 (1.1–1.1)	7.3
X59 Exposure to unspecified factor	9415	0.9 (0.9–1.0)	6.7
A41 Other sepsis	6574	0.7 (0.6–0.7)	4.7
N19 Unspecified kidney failure	6173	0.6 (0.6–0.6)	4.4
R99 Unknown cause of death	5966	0.6 (0.6–0.6)	4.2
I10 Essential hypertension	5409	0.5 (0.5–0.6)	3.8
B99 Unspecified infectious diseases	4188	0.4 (0.4–0.4)	3.0
I70 Atherosclerosis	3731	0.4 (0.4–0.4)	2.6
**Minor GC**	*149,665*	*15.0 (14.9–15.0)*	
I64 Unspecified stroke	43,814	4.4 (4.3–4.4)	29.3
J18 Unspecified pneumonia	41,753	4.2 (4.1–4.2)	27.9
C80 Malignant neoplasm, unknown primary site	11,013	1.1 (1.1–1.1)	7.4
E14 Unspecified diabetes mellitus	10,425	1.0 (1.0–1.1)	7.0
I69 Sequelae of cerebrovascular disease	10,124	1.0 (1.0–1.1)	6.8
I51 Ill-defined heart disease	8673	0.9 (0.8–0.9)	5.8
I49 Unspecified cardiac arrythmia	1981	0.2 (0.2–0.2)	1.3
C91 Lymphoid leukemia (unspecified)	1919	0.2 (0.2–0.2)	1.3
I42 Unspecified cardiomyopathy	1906	0.2 (0.2–0.1)	1.3
C26 Malignant neoplasm of ill-defined digestive organs	1544	0.2 (0.1–0.2)	1.0

Data source: NCoDR

We found another spectrum of garbage codes in the young. For deaths in the 15–49 years age group, three groups of accidental poisonings (X42, X44, and X41) accounted for 53.2% of the major garbage codes, and F19 (unspecified drug abuse) for another 8.1%.

There were also differences according to the place of death, especially for major garbage codes. In hospitals, the most common major garbage codes were I50 (heart failure) and A41 (other sepsis), in nursing homes I50 (heart failure) and R54 (senility). In deaths outside health care institutions, R99 (unknown cause of death), R96 (sudden death), I46 (cardiac arrest), I50 (heart failure) and X42 (accidental poisoning with narcotic or psychodysleptics) were common. The most common minor garbage codes were I64 (unspecified stroke) and J18 (unspecified pneumonia) in deaths at hospitals and nursing homes, whereas I51 (ill-defined heart disease), and I64 (unspecified stroke) were commonly used in deaths occurring outside health care facilities.

Detailed tables are presented in the supplemental material, Tables S2a-d.

### Garbage codes over time

For major garbage codes, there were fluctuations over time, with an increasing tendency overall and a peak in 2013. In the first four years of the study period (1996–1999), the proportions of deaths coded with a major garbage code were 13.5% in women, 9.9% in men. In the last five years (2015–2019), the proportions were 15.3% in women, 12.5% in men.

**Fig. 1:**
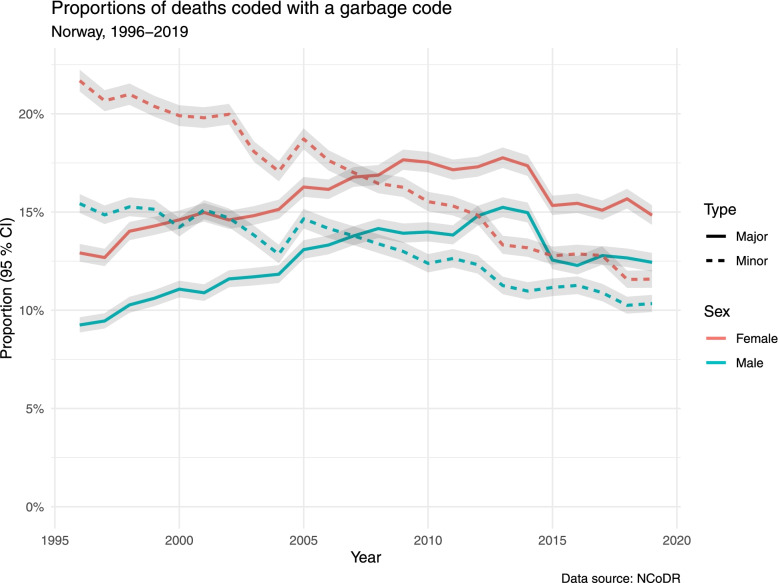
Proportions of deaths coded with a garbage code

A reduction in the proportion of deaths coded with minor garbage codes was found for both sexes. In the first four years of the study period, the proportions were 20.9% in women, 15.2% in men. In the last five years, the proportions were 12.3% in women, 10.8% in men (Fig. 1, Table 2).

**Table 2:** Garbage codes in Norway 1996–2019, according to sex and time period

*Women*					
**Year**	**All deaths**	**Major garbage codes**		**Minor garbage codes**	
	N	N	Percent (95% CI)	N	Percent (95% CI)
1996–1999	88,601	11,947	13.5 (13.3–13.7)	18,538	20.9 (20.7–21.2)
2000–2004	109,982	16,302	14.8 (14.6–15.0)	20,889	19.0 (18.8–19.2)
2005–2009	106,496	17,836	16.7 (16.5–17.0)	18,329	17.2 (17.0–17.4)
2010–2014	105,564	18,391	17.4 (17.2–17.7)	15,251	14.4 (14.2–14.7)
2015–2019	103,208	15,769	15.3 (15.1–15.5)	12,719	12.3 (12.1–12.5)
*Total*	*513,851*	*80,245*	*15.6 (15.5–15.7)*	*85,725*	*16.7 (16.6–16.8)*
*Men*					
**Year**	**All deaths**	**Major garbage codes**		**Minor garbage codes**	
	N	N	Percent (95% CI)	N	Percent (95% CI)
1996–1999	88,442	8755	9.9 (9.7–10.1)	13,418	15.2 (14.9–15.4)
2000–2004	104,420	11,915	11.4 (11.2–11.6)	14,794	14.2 (14.0–14.4)
2005–2009	98,682	13,476	13.7 (13.4–13.9)	13,616	13.8 (13.6–14.0)
2010–2014	98,083	14,288	14.6 (14.3–14.8)	11,688	11.9 (11.7–12.1)
2015–2019	96,650	12,125	12.5 (12.3–12.8)	10,423	10.8 (10.6–11.0)
*Total*	*486,277*	*60,557*	*12,5 (12.4–12.5)*	*63,939*	*13.1 (13.1–13.2)*

Data source: NCoDR

### Change in pattern of garbage codes

No single pattern explained the change in the proportion of deaths with a major garbage code. The slow increase to 2013 and the subsequent decline was the sum of multiple smaller changes, both increases and declines. There was an increase of X59 deaths (exposure to unspecified factor) from 0.2% in the first four years to 1.6% in 2010–2014, and a decline to 1.0% in 2015–2019. The B99 deaths (unspecified infectious diseases) increased from 0.1 to 0.9% during the study period. There were also increases in A41 (other sepsis) and R99 (unknown cause of death). The proportion of deaths coded with I50 (heart failure) declined from 4.2% in the first four years to 3.0% in the last five year.

The reduction of minor garbage codes was almost fully accounted for by decline in I64 (unspecified stroke), 6.5% in the first four years, 2.4% in the last five years, and J18 (unspecified pneumonia), decline from 4.6 to 3.6%. The changes in these two codes alone explained 80% of the reduction.

I50 (heart failure), X59 (exposure to unspecified factor), X42 (accidental poisoning by narcotics and psychodysleptics) and I64 (unspecified stroke) are discussed more thoroughly below. Some of the observed changes (notably in X59 and accidental poisonings) can be explained by changes in the coding rules.

### Sex and age

A larger proportion of all deaths in women were coded with a garbage code, both major and minor. For major garbage codes the proportions were 15.6% in women, 12.5% in men. For minor garbage codes: 16.7% in women, 13.1% in men. The sex difference decreased towards the end of the study period (Fig. 1). When comparing age-adjusted proportions, there was hardly any difference between sexes in the last 5-year period. Major garbage codes: 14.0% in women, 13.5% in men; minor garbage codes: 11.5% in women, 11.8% in men (supplemental Fig. S1).

**Fig. 2:**
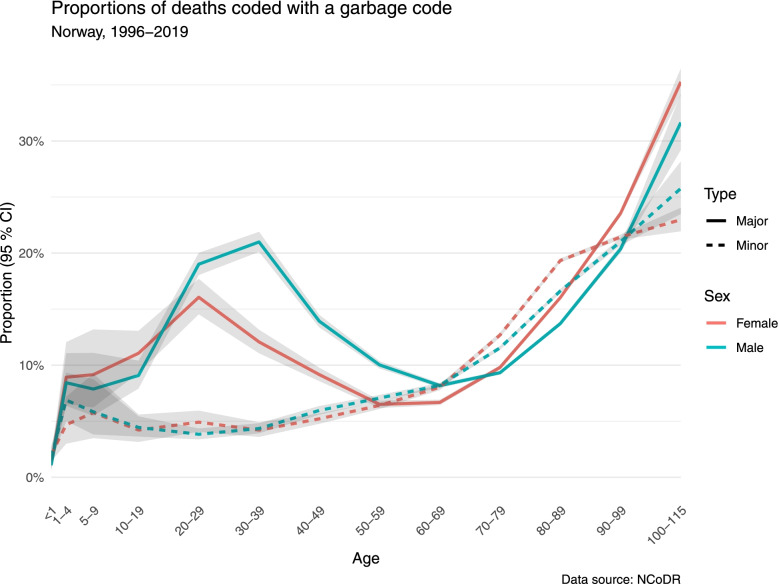
Proportions of deaths coded with a garbage code, according to age and sex

The proportion of deaths with a garbage code rose with age at death above circa 60 years. In the group with age at death ≥90 years, 24.1% of women and 20.6% of men had a major garbage code and 21.5% of women and 21.1% of men a minor garbage code. Major garbage codes were also used in a high proportion of deaths in young adults. Within each age segment, there are relatively small differences between men and women, except for major garbage codes in young adults (Fig. 2, Table 3).

**Table 3:** Garbage codes in Norway 1996–2019, according to sex and age

	All deaths	Major garbage codes		Minor garbage codes	
	N	N	Percent (95% CI)	N	Percent (95% CI)
**Women**					
Under 1 year	1852	20	1.1 (0.7–1.7)	37	2.0 (1.4–2.8)
1–4 years	448	40	8.9 (6.5–12.1)	21	4.7 (3.0–7.2)
5–14 years	628	70	11.1 (8.9–13.9)	29	4.6 (3.2–6.7)
15–49 years	15,893	1723	10.8 (10.4–11.3)	778	4.9 (4.6–5.2)
50–79 years	157,436	13,401	8.5 (8.4–8.7)	16,662	10.6 (10.4–10.7)
80–89 years	202,338	32,419	16.0 (15.9–16.2)	39,113	19.3 (19.2–19.5)
90 years and above	135,256	32,572	24.1 (23.9–24.3)	29,086	21.5 (21.3–21.7)
*Total*	*513,851*	*80,245*	*15.6 (15.5–15.7)*	*85,726*	*16.7 (16.6–16.8)*
**Men**					
Under 1 year	2412	26	1.1 (0.7–1.6)	44	1.8 (1.3–2.5)
1–4 years	581	49	8.4 (6.4–11.1)	40	6.9 (5.0–9.3)
5–14 years	825	72	8.7 (6.9–10.9)	43	5.2 (3.8–7.0)
15–49 years	31,017	5125	16.5 (16.1–16.9)	1559	5.0 (4.8–5.3)
50–79 years	227,868	20,713	9.1 (9.0–9.2)	22,544	9.9 (9.8–10.0)
80–89 years	166,886	22,875	13.7 (13.5–13.9)	27,731	16.6 (16.4–16.8)
90 years and above	56,688	11,699	20.6 (20.3–21.0)	11,978	21.1 (20.8–21.5)
*Total*	*486,277*	*60,559*	*12.5 (12.4–12.5)*	*63,939*	*13.1 (13.1–13.2)*

Data source: NCoDR

### Place of death

The age-adjusted proportion of deaths coded with a major garbage code was lowest for deaths in hospitals and in nursing homes and other health care institutions, and highest in deaths occurring outside health care facilities: at home, in other known locations, and where the place of death was unregistered. For minor garbage codes, the age-adjusted proportion was highest in nursing homes (Table 4).

**Table 4:** Garbage codes in Norway 1996–2019, according to place of death

Place of death	All deaths		All garbage codes			Major garbage codes			Minor garbage codes		
	N	Median age at death (years)	N	Unadjusted (%)	Age adjusted (%) (95% CI)	N	Unadjusted (%)	Age adjusted (%) (95% CI)	N	Unadjusted (%)	Adjusted (%) (95% CI)
Hospital	366,855	78	74,666	20.4	23.7 (23.5–23.9)	33,531	9.1	11.0 (10.9–11.2)	41,135	11.2	12.7 (12.6–12.9)
Nursing home	434,271	86	157,632	36.3	31.6 (31.4–31.8)	68,638	15.8	13.6 (13.4–13.7)	88,994	20.5	18.0 (17.9–18.2)
At home	149,203	75	44,238	29.6	34.3 (34.0–34.7)	28,545	19.1	22.0 (21.7–22.3)	15,693	10.5	12.3 (12.1–12.6)
Other known	34,620	61	9037	26.1	34.3 (33.2–35.5)	6796	19.6	24.0 (23.0–24.9)	2241	6.5	10.3 (9.7–11.0)
Not known	15,179	73	4896	32.3	36.3 (35.2–37.5)	3294	21.7	23.1 (22.2–24.1)	1602	10.6	13.2 (12.5–13.9)
*Total*	*1,000,128*	*81*	*290,469*	*29.0*	*30.0 (29.9–30.2)*	*140,804*	*14.1*	*14.7 (14.6–14.8)*	*149,665*	*15.0*	*15.4 (15.3–15.4)*

Data source: NCoDR

### Autopsy

During the study period, 4.2% of the deceased underwent a forensic autopsy, 4.7% a non-forensic one. The median age of death was 51 years in the forensic autopsy group, 72 years in the non-forensic autopsy group and 82 years in the not autopsied. More deceased men than women underwent an autopsy, both forensic and non-forensic (6.2% vs 2.4% and 5.6% vs 3.8%).

The relationship between garbage codes and autopsy showed a mixed pattern. In deceased undergoing a non-forensic autopsy, the age-adjusted proportion of deaths coded with a major garbage code (7.3%) was lower than in the non-autopsied (14.6%). In deceased undergoing a forensic autopsy, the age-adjusted proportion of major garbage codes was almost the same as in the non-autopsied, (12.8%). In both types of autopsy, the age-adjusted proportions of minor garbage codes were lower than in the non-autopsied (forensic 5.8%, non-forensic 6.8%, non-autopsied 15.8%) (Table 5).

**Table 5:** Garbage codes in Norway 1996–2019, according to autopsy type

Autopsy type	All deaths			Major garbage codes			Minor garbage codes		
	N	Percent (95% CI)	Median age at death (years)	N	Unadjusted (%)	Age adjusted (%) (95% CI)	N	Unadjusted (%)	Age adjusted (%) (95% CI)
Forensic autopsy	42,074	4.2 (4.2–4.2)	51	6953	16.5	12.8 (11.6–14.2)	1732	4.1	5.8 (4.9–6.8)
Non-forensic autopsy	46,862	4.7 (4.6–4.7)	72	3118	6.7	7.3 (6.9–7.8)	2634	5.6	6.8 (6.4–7.3)
No autopsy	911,192	91.1 (91.1–91.2)	82	130,733	14.3	14.6 (14.5–14.7)	145,299	15.9	15.8 (15.7–15.9)
*Total*	*1,000,128*		*81*	*140,804*	*14.1*	*14.7 (14.6–14.8)*	*149,665*	*15.0*	*15.4 (15.3–15.4)*

Data source: NCoDR

### Factors correlating with use of a garbage code

We performed logistic regression analyses to investigate the determinants of use of a garbage code as the underlying cause of death. All the investigated factors had a significant explanatory effect, both in single-predictor and multiple-predictor models. When comparing the odds ratios for deaths coded with a garbage code, we noticed that the sex difference was less pronounced, and that the odds ratio for deaths in nursing homes was lower in the multiple-predictor model than in the single-predictor model. For deaths occurring in other known places, the odds ratio was higher in the multiple-predictor than in the single-predictor models. For deceased that underwent autopsy, the odds ratios were also higher in the multiple-predictor model compared to the single-predictor model. In the multi-predictor model, the most important explanatory factors (evaluated by ranking of the LR statistic) were age and place of death (Table 6).

**Table 6:** Logistic regression analysis of determinants for use of garbage codes in Norway 1996–2019

Explanatory variable	All GC (%) ***N*** = 290,469	All deaths ***N*** = 1,000,128	Single predictor models				Multiple predictor model			
			OR	(95% CI)	LR stat*	*p* value	OR	(95% CI)	LR stat*	*p* value
**Year of death**					**1645**	**< 0.001**			**3644**	**< 0.001**
1996–1999	52,658 (29.7)	177,043	1 (ref.)				1 (ref.)			
2000–2004	63,900 (29.8)	214,402	1.00	(0.99–1.02)			0.96	(0.95–0.98)		
2005–2009	63,257 (30.8)	205,178	1.05	(1.04–1.07)			0.96	(0.95–0.98)		
2010–2014	59,618 (29.3)	203,647	0.98	(0.96–0.99)			0.85	(0.84–0.86)		
2015–2019	51,036 (25.5)	199,858	0.81	(0.81–0.82)			0.68	(0.67–0.69)		
**Sex**					**5454**	**< 0.001**			**482**	**< 0.001**
Female	165,971 (32.3)	513,851	1 (ref.)				1 (ref.)			
Male	124,498 (25.6)	486,277	0.72	(0.72–0.73)			0.90	(0.89–0.91)		
**Age at death**					**48,158**	**< 0.001**			**30,379**	**< 0.001**
Under 1	127 (2.98)	4264	1 (ref.)				1 (ref.)			
1–4	150 (14.6)	1029	5.56	(4.34–7.13)			4.57	(3.56–5.86)		
5–14	214 (14.7)	1453	5.63	(4.48–7.09)			4.44	(3.54–5.60)		
15–49	9185 (19.6)	46,910	7.93	(6.67–9.52)			5.87	(4.93–7.05)		
50–79	73,320 (19.0)	385,304	7.66	(6.45–9.18)			5.81	(4.89–6.97)		
80–89	122,138 (33.1)	369,224	16.1	(13.6–19.3)			11.3	(9.49–13.5)		
90–115	85,335 (44.5)	191,944	26.1	(22.0–31.3)			17.4	(14.6–20.9)		
**Place of death**					**25,291**	**< 0.001**			**10,369**	**< 0.001**
Hospital	74,666 (20.4)	366,855	1 (ref.)				1 (ref.)			
Nursing home	157,632 (36.3)	434,271	2.23	(2.21–2.25)			1.62	(1.60–1.64)		
At home	44,238 (29.6)	149,203	1.65	(1.63–1.67)			1.76	(1.73–1.78)		
Other known	9037 (26.1)	34,620	1.38	(1.35–1.42)			1.84	(1.79–1.89)		
Unknown	4896 (32.3)	15,179	1.86	(1.80–1.93)			2.08	(2.00–2.15)		
**Autopsy**					**9761**	**< 0.001**			**1828**	**< 0.001**
No autopsy	276,032 (30.3)	911,192	1 (ref.)				1 (ref.)			
Non-forensic	5752 (12.3)	46,862	0.32	(0.31–0.33)			0.56	(0.55–0.58)		
Forensic	8685 (20.6)	42,074	0.60	(0.58–0.61)			0.83	(0.81–0.85)		

Data source: NCoDR

*LR stat:* Likelihood ratio statistic (−2LogL)

Results from separate analyses for major and minor garbage codes are presented in the supplemental material, Tables S1a-b. For major garbage codes, the most important explanatory factors were age and place of death, whereas for minor garbage codes, also the year of death was one of the most important factors.

### Other registered diagnoses in deaths coded with a garbage code

Of the deaths coded with a major or minor garbage code as the underlying cause of death, 104,680 of 290,469 (36.0, 95% CI 35.9–36.2%) had one or more non-garbage codes among the registered diagnoses. The proportion varied considerably between different places of death: hospital 44.4% (44.1–44.8%), nursing home 37.0% (36.8–37.3%), at home 23.8% (23.8–24.2%), other known place 15.5% (14.8–16.3%), and 25.5% (24.3–26.7%) where the place of death was unknown.

Grouped according to the GBD cause list (level 3), the most prevalent non-garbage codes were Alzheimer disease and other dementias (24.1% of the cases with at least one non-garbage code), ischaemic heart disease (17.8%), atrial fibrillation and flutter (11.2%), chronic obstructive pulmonary disease (COPD) (8.1%), and urinary tract infection (6.0%). There were only small differences in rank between the groups with major and minor garbage codes, but different garbage codes had very different patterns of non-garbage codes. (Supplementary Tables S3a and b show the most common non-garbage codes for each of the most prevalent major and minor garbage codes).

### More on the most prevalent garbage codes

#### I50 heart failure

I50 (heart failure) is the most prevalent major garbage code in Norway, 3.7% of all deaths in the study period. The proportion of deaths coded with I50 declined from 4.2% (95% CI 4.1–4.3%) in the first 4 years to 3.0% (2.9–3.1%) in the last five years. In the same years, the proportion of deaths coded to cardiovascular causes except cerebrovascular disease, declined from 32.6 to 20.6%.

Of the deaths coded with I50 as the underlying cause of death, 12,844 of 36,683 (35.0, 95% CI 34.5–35.5%) had one or more non-garbage codes among the registered diagnoses. The most prevalent were: Alzheimer disease and other dementias, chronic obstructive pulmonary disease, atrial fibrillation and flutter, urinary diseases and stroke.

#### I64 unspecified stroke

I64 (unspecified stroke) is the most prevalent minor garbage code, found in 4.4% of all deaths, and there has been a decline in the proportion of cases from 6.7% (95% CI 6.6–6.9%) of all deaths in the first four years to 2.4% (2.3–2.5%) in the last five years. At the same time, there has been a decline in the proportion of deaths due to all cerebrovascular diseases (I60-I69) from 11.3 to 5.9%. The proportion of all cerebrovascular diseases coded to unspecified stroke declined from 59.5 to 40.7% during the study period.

Of the deaths coded with I64 as the underlying cause of death, 18,156 of 43,814, (41.4, 95% CI 40.0–41.9%) had one or more non-garbage codes among the registered diagnoses. The five most prevalent non-garbage codes were Alzheimer disease and other dementias, ischaemic heart disease, atrial fibrillation and flutter, (specified) stroke, and chronic obstructive pulmonary disease.

#### X42 accidental poisoning by narcotics and psychodysleptics

X42 (accidental poisoning by narcotics and psychodysleptics) is the most prevalent garbage code in the NCoDR for the age group 15–49 years, found in 5.0% (95% CI 4.8–5.2%) of all deaths and constituting 34.3% of all major garbage codes in this age group. The three accidental poisonings codes X41, X42, and X44 together account for 53.2% of all major garbage codes in this age group (Supplemental Table S2b). There is a striking time trend, with a mean number of 16 yearly cases in the years 1996–2002, and a mean number of 165 yearly cases in the years 2003–2019. The same codes explain the high proportion of major garbage codes in forensic autopsies (supplementary Table S2d). Before 2003, an accidental drug poisoning in a person with addiction was coded as a disorder due to substance use (ICD-10 section F11–16, F19). In 2003, there was a change in the rules from the WHO, and accidental poisonings were to be coded as external causes of death (ICD-10 section X40-X49). Most codes in this section are regarded as garbage codes by the GBD, whereas many of the corresponding codes in the section of the F chapter are not.

#### X59 exposure to unspecified factor

X59 (exposure to unspecified factor) is the most prevalent garbage code in the external cause of death section, found in 0.9% of all deaths. Also here, there is a striking time trend, with a mean of 95 yearly cases in 1996–2004, a mean of 644 yearly cases 2005–2014, a drop to 264 and 232 cases in 2015 and 2016, and then again a rise to a mean of 540 cases 2017–2019. Before 2005, a local guideline in NCoDR stated that deaths from fractures of the femur without information on the circumstances were to be coded as W19 (accidental fall), which is not regarded as a garbage code by the GBD. From 2005 and onward, NCoDR adhered to the WHO rules, coding these cases as X59. In the years 2015 and 2016, a quality improvement project in the NCoDR caused a temporary fall in the number of X59 cases [18].

Of the deaths coded with X59 as the underlying cause of death, 6442 of 9415, (68.4, 95% CI 67.5–69.3%) had one or more non-garbage codes among the registered diagnoses. The most prevalent were: Effects of medical treatment, Alzheimer disease and other dementias, ischaemic heart disease, atrial fibrillation and flutter, and chronic obstructive pulmonary disease. The coding of “effects of medical treatment” does not necessarily indicate a complication, only that some kind of medical or surgical procedure was mentioned on the death certificate. The nature of injury (S- and T-codes in ICD-10), is by definition a garbage code and therefore not counted among the non-garbage codes. In 69.8% of the X59 deaths, fracture of femur (S72.X) was registered as the nature of injury.

## Discussion

In this population-based study, we used data from the Norwegian Cause of Death Registry for the years 1996–2019 to investigate the use of garbage codes for the underlying cause of death. We found that the proportion of deaths coded with major garbage codes increased slightly during the study period, whereas the proportion of minor garbage codes declined. The two most important determinants of use of garbage codes in the registry were the age of the deceased and the place of death.

### Strengths and limitations

The data material is large and comprehensive, and consists of all deaths in Norway with a registered cause of death (98.7% of all deaths) over a 24-year period. ICD-10 has been used as classification system throughout the period, and data processing and coding in the registry has been performed by skilled personnel in Statistics Norway up to 2013 and at The Norwegian Institute of Public Health from 2014.

During the study period, there has been some changes in the coding rules, notably for external causes of death. This is reflected in some of the time trends, for example for the major garbage codes X42 (accidental poisoning by narcotics and psychodysleptics) and X59 (exposure to unspecified factor).

We have used the list of garbage codes from the GBD Study, as we believe that much of the current research on the quality of cause of death statistics is linked to the GBD. The composition of this list is based upon choices made by the GBD research team, and there has been a gradual development over the iterations of the GBD analyses [5, 7].

The list of garbage codes from the GBD is much longer than the list of ill-defined causes of death from the WHO [3]. The results of this study would be different if we had used another definition of garbage codes. Use of the GBD list is both a strength and a weakness. It makes it possible to compare our results with other studies that use the GBD framework, but makes it difficult to compare with studies using another definition.

A garbage code may arise on several stages in the diagnostic, certification, and coding process of deaths, and knowing the contribution of each stage could guide quality improvement efforts. A weakness in our study is that we cannot discern the importance of each stage.

We have investigated the correlation of a number of putative explanatory factors with the use of garbage codes, but there are likely also other important factors, not included in our analyses.

If a death is coded with a non-garbage code as the underlying cause of death, it does not imply that the cause of death is correct. An example: the symptoms of a perforated peptic ulcer (a valid diagnosis) might be misinterpreted as a myocardial infarction (another valid diagnosis). This study was not designed to ascertain the magnitude of incorrect diagnoses.

The study is from a single country, and from the ICD-10 period only. Therefore, we cannot claim that the results can be generalized to other countries.

### Discussion of results

#### General considerations

We found that 29.0% of the deaths in Norway in the study period were coded with a garbage code as the underlying cause of death, 14.1% major and 15.0% minor. It is the use of major garbage codes that are considered most deleterious for public health analyses, as they convey least information. Worldwide, the proportion of major garbage codes in cause of death registries ranges from 4% to more than 80% in the latest available year [7]. The proportion in Norway is similar to several comparable countries, such as Sweden (13% in 2017), Denmark (16% in 2015), Germany (15% in 2016), and the Netherlands (16% in 2016), but higher than e.g. Finland (6% in 2016), UK (9% in 2017), and New Zealand (4% in 2015). This would suggest that even if Norway is in the lower end, there is still potential for improvement.

#### Age and sex

We found that the proportion of deaths assigned a garbage code increased with the age of the deceased, and hence were larger in women than in men, as median age of death was 6 years higher in women. Other studies have divergent observations. Iburg et al. [2] found that in most of the 20 studied countries, there were no large age gradient in major garbage codes. Johnson et al. [6] stated that the garbage code proportion often is higher in locations with an elderly population, and suggest using age standardization to improve comparability. An age gradient has been described in Greenland [19], Brazil [20], and Korea [21]. Flagg and Anderson [22] found an age gradient in the Unites States, but they used another definition of unsuitable causes of death. Adair et al. [23] found a slightly higher age-adjusted garbage code proportion in women in a study on data from 42 countries.

Older people often have several diseases, and it can be challenging to identify a single cause of death. One could also speculate that as the end of life comes closer, the focus of the health care can be more on symptom relief than on identifying and treating the exact cause. There was a large proportion of deaths with a major garbage code in the 15–49 year group. This can almost fully be explained by coding of accidental poisonings, discussed more closely below.

#### The place of death

The proportion of deaths assigned a garbage code was lowest in hospitals, this can probably be explained by on one hand better diagnostic resources in hospitals and on the other hand more sudden, unexpected or unattended deaths outside health care institutions. The risk of dying before reaching hospital is higher in sudden catastrophic illness. This is reflected in the spectrum of major garbage codes for deaths outside health care institutions, with R96 (sudden death), R99 (unknown cause of death), and I46 (cardiac arrest) among the most prevalent. The unadjusted proportion was highest in nursing homes, but the age-adjusted proportions were lower than deaths in other places, except in hospitals, reflecting the high median age of death in nursing homes.

### The origin of garbage codes

We believe that a garbage code for the cause of death can arise in two fundamentally different ways: either by insufficient diagnosis, or by faults in the certification and coding process. An insufficient diagnosis is when the certifying doctor does not have enough information on the real underlying cause of death. A typical example is if a person is found dead and no autopsy is performed. Even if the medical doctor is confident in the principles regarding certification of death, it is not possible to give an informative diagnosis.

A fault in certification is when the certifying doctor possesses enough information to give a sufficient cause of death, but because of lack of training or otherwise fails to give a proper statement on the cause of death. The second instance, but not the first, has a potential of improvement by better training and information on how to certify a death. From our study, we cannot distinguish between these two origins. However, the presence of non-garbage codes on the death certificate in a case could perhaps be an indication that there is more information available. We found that the proportion of garbage code deaths with non-garbage codes in the records was considerably lower in deaths outside health care institutions. One cannot claim, however, that the true underlying cause of death is among these non-garbage codes.

The central coding in the registry can also influence the prevalence of garbage codes in the cause-of-death statistics, for instance by asking for additional information in unclear cases.

The frequency and type of garbage codes can be influenced by several factors beside the certifying doctor’s abilities.

#### The epidemiological situation

If there is a rise or decline in diseases that might give origin to a certain garbage code on the death certificate, this might lead to a corresponding rise or decline in the number of deaths coded with this code. The mortality of cardiovascular diseases have declined in Norway [24], and in parallel with this the proportion of all deaths coded with garbage codes related to these causes of death, such as I50 (heart failure) and I64 (unspecified stroke).

#### The diagnostic efforts: pre- and postmortem

If the diagnostic process before the death of a patient has been comprehensive, there is more information in the records that can be used to give a specific cause of death. Stroke is a good example. The reduction in the number of deaths coded with I64 (unspecified stroke) is larger than can be explained by the changing epidemiological situation alone. The fraction of all cerebrovascular deaths coded with I64 has declined from almost 60 to 40% during the study period. The more widespread use of diagnostic procedures to distinguish between thrombotic and haemorrhagic stroke [25] can probably explain more specific causes of death.

The age-adjusted proportion of deaths assigned a garbage code is generally lower in the autopsied than in the non-autopsied. The relatively high proportion of major garbage codes in the persons undergoing forensic autopsy can be explained by accidental poisonings (see below).

#### The WHO coding rules and local guidelines

In 2003, there was a change in the coding rules from WHO; accidental poisonings with drugs of abuse should be coded with external causes of death (ICD-10 X41, X42 and X44) instead of deaths due to drug abuse (F11–16, F19) [26]. GBD views most of the codes in X40-X49 as garbage codes and most codes in F10-F19 as non-garbage codes. Following the WHO guidelines thus leads to more use of garbage codes. The reason that the codes for accidental poisonings are regarded as garbage codes are mainly that GBD considers drug overdoses as dependency disorder-related deaths and also that some of these deaths in reality are suicides and thus should be redistributed (M. Naghavi, IHME, personal communication).

Before 2005, fractures of the femur without information on the circumstances around the injury were by default coded as W19 (accidental falls). From 2005, these deaths were coded with X59 (exposure to unidentified factor), a major garbage code [26]. More diligently following the WHO coding rules lead to a rise in the number of garbage codes. We have earlier analysed these deaths in detail [18]. Most of these cases really are accidental falls, but many of the deaths occur a long time after the incident, and the certifying doctor may not know anything about the circumstances or is unfamiliar with the rules for coding of external cause deaths. The official Norwegian online coding tool [27], used by the certifying medical doctors, is not fully congruent with the international version of ICD-10 for external causes, placing more emphasis on the nature of injury and less on the circumstances.

### Other diagnoses present on the death certificate

We found that 36% of the garbage coded deaths had other, non-garbage codes mentioned on the death certificate. Could these diagnoses have been used in a multiple cause of death approach to identify a more informative underlying cause of death? There are at least two objections to this: First, the NCoDR, as other cause of death registries, must conform to the rules and guidelines from the WHO. “Garbage code” is not an ICD-10 concept. The closest is the lists of ill-defined conditions and conditions unlikely to cause death, mentioned in the introduction (Annex 7.3 and 7.4 in the instruction manual of ICD-10 [3]). They are to be avoided, if possible (Step SP7 and SP8 in the ICD-10 coding rules). If a more specific code is present somewhere on the death certificate, it should be selected as the underlying cause of death. This can be seen in that a non-garbage code is mentioned in only 2.3% of the deaths coded with R96 (sudden death) as underlying cause of death (Supplemental Table S3a). The list of garbage codes from the GBD is much more extensive than the list of ill-defined codes from WHO. As the NCoDR must follow the WHO rules and guidelines, codes that are accepted by WHO, but classified as garbage codes by the GBD, cannot be disregarded. Second, presence of a non-garbage code can give valuable information on co-morbidity, but not necessarily on the real underlying cause of death. For example, for the minor garbage code C80 (malignant neoplasm, site unknown), the most commonly occurring non-garbage code is ischaemic heart disease, which does not point to a specific origin of cancer (Supplemental Table S3b). In other instances, one may find a candidate for the underlying cause of death among the non-garbage codes. For example, the most common non-garbage code for J18 (unspecified pneumonia) is Alzheimer disease and other dementias (Supplemental Table S3b), and this could in some instances be the condition leading to an airway infection.

### Implications of the study

If a large proportion of the deaths in a population is assigned a garbage code for the underlying cause of death, the cause of death data would be less useful for public health purposes such as health surveillance, analyses and research. We have found that the most important determinants of use of garbage codes as underlying cause of death is advanced age at death and place of death outside hospital. Knowledge of the national epidemiological situation as well as the rules and guidelines for mortality coding is essential for understanding the prevalence and distribution of garbage codes.

Better training of the certifying medical doctors could probably eliminate some of the garbage codes that are caused by certification errors [28], but not those that are caused by lack of information. The Norwegian Medical Association already provides an online tutorial on death certification [29], and this could be made compulsory, at least for doctors who regularly completes death certificates. Alfsen and Lyckander [30] found that the cause of death could be changed in 18% of the deaths in a Norwegian hospital just from better use of the information in the patient records and adherence to the certification guideline, but this study was not directed against garbage codes. Many of the deaths outside hospitals are certified by doctors on call who not necessarily have access to the medical records of the deceased. Better access to relevant information, for example via the summary care record [31] would probably be useful, as well as more use of autopsy [32]. In Norway, the majority of deaths occur in health care institutions, and almost all are certified by a medical doctor. Verbal autopsy (in the original sense with interview of the relatives of the deceased or other lay persons) seems less relevant for Norwegian and similar high-income countries, but might be of value in low-resource settings.

Directed quality assurance efforts at the cause of death registries with queries to the certifying doctors can improve the data quality [18].

From the year 2022, electronic certification of deaths is compulsory in Norway [33]. In our study, only 0.13% of the total number of deaths were electronically certified. From 2022, all deaths will be certified by this system. Time will show whether this has influence on the data quality. In an electronic system, there is a potential for decision support or real-time feedback for the certifying medical doctors, for instance discouraging the use of garbage codes. The present-day system for electronic certification of deaths in Norway has only limited decision support and does not give feedback to the physician on use of garbage codes [33].

Issuing death certificates is a professional duty for the individual medical doctor. To ensure conformity in practice among different practitioners, there thus should be some kind of collegial or institutional mechanisms for quality assurance of this work. The proportion of garbage codes in an otherwise well working system of death certification, as in Norway, may indicate that there still is a considerable room for further improvement.

## Supplementary Information

**Additional file 1.**

## Data Availability

According to Norwegian data privacy regulations, it is not possible to make the data publicly available. Researchers wishing to replicate or expand the study may seek approval by the Committee for Medical Research Ethics and request data from the Norwegian Institute of Public Health.

## Abbreviations

WHO: World Health Organization
ICD-10: International Statistical Classification of Diseases and Related Health Problems, 10th Revision
GBD: The Global Burden of Disease
GC: Garbage code (as defined by the Global Burden of Disease)
NCoDR: The Norwegian Cause of Death Registry at the Norwegian Institute of Public Health
Q1-Q3: The range from the 25th to the 75th percentile
CI: Confidence interval
