# Supplementary material for: Garbage codes in the Norwegian Cause of Death Registry 1996–2019
Source: BMC Public Health. 2022 Jul 7;22:1301. doi: 10.1186/s12889-022-13693-w (PMC9261062; doi:10.1186/s12889-022-13693-w)
Supplement: Supplementary file 1 — Additional file 1. [file 12889_2022_13693_MOESM1_ESM.pdf]

Non-informative codes in the Norwegian Cause of Death Registry – supplemental material

Figure S1 – Age adjusted proportions of deaths coded with a garbage code

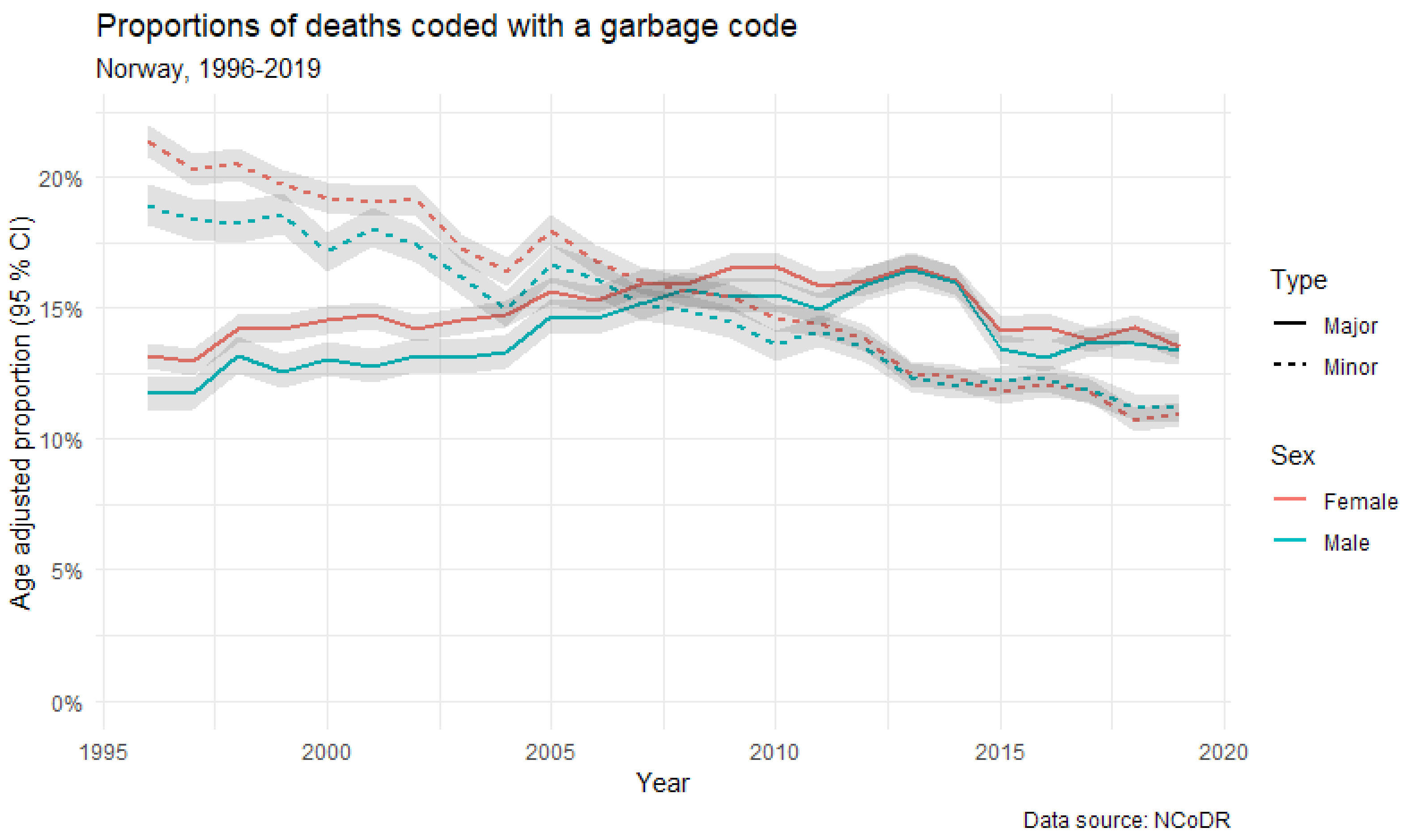

Table S1a – Logistic regression, factors correlated with major garbage codes

Explanatory variable	Major GC (%) N = 153,426	All deaths N = 1,000,128	Single predictor models				Multiple predictor model			
			OR	95 % CI	LR stat*	p	OR	95 % CI	LR stat*	p
**Year of death**					**1,891**	**< 0.001**			**1,158**	**< 0.001**
1996-1999	20,702 (11.7)	177,043	1 (ref.)				1 (ref.)			
2000-2004	28,217 (13.2)	214,402	1.14	(1.12-1.17)			1.12	(1.09-1.14)		
2005-2009	31,312 (15.3)	205,178	1.36	(1.33-1.39)			1.28	(1.26-1.31)		
2010-2014	32,679 (16.0)	203,647	1.44	(1.44-1.42)			1.32	(1.29-1.34)		
2015-2019	27,894 (14.0)	199,858	1.22	(1.20-1.25)			1.10	(1.08-1.12)		
**Sex**					**2,073**	**< 0.001**			**409**	**< 0.001**
Female	80,245 (15.6)	513,851	1 (ref.)				1 (ref.)			
Male	60,559 (12.5)	486,277	0.77	(0.76-0.78)			0.88	(0.87-0.89)		
**Age at death**					**22,157**	**< 0.001**			**19,019**	**< 0.001**
Under 1	46 (1.1)	4,264	1(ref.)				1 (ref.)			
1-4	89 (8.7)	1,029	8.68	(6.07-12.6)			6.35	(4.44-9.22)		
5-14	142 (9.8)	1,453	9.93	(7.14-14.1)			6.96	(5.00-9.88)		
15-49	6848 (14.6)	46,910	15.7	(11.9-21.3)			9.42	(7.12-12.8)		
50-79	34,114 (8.9)	385,304	8.91	(6.75-12.1)			7.17	(5.42-9.73)		
80-89	55,294 (15.0)	369,224	16.2	(12.2-21.9)			13.8	(10.4-18.7)		
90-115	44,271 (23.1)	191,944	27.5	(20.8-37.3)			22.8	(17.3-31.0)		
**Place of death**					**13,649**	**< 0.001**			**11,757**	**< 0.001**
Hospital	33,531 (9.1)	366,855	1 (ref.)				1 (ref.)			
Nursing home	68,638 (15.8)	434,271	1.87	(1.84-1.89)			1.31	(1.30-1.33)		
At home	28,545 (19.1)	149,203	2.35	(2.31-2.39)			2.45	(2.40-2.49)		
Other known	6796 (19.6)	34,620	2.43	(2.36-2.50)			2.90	(2.81-3.00)		
Unknown	3294 (21.7)	15,179	2.76	(2.65-2.87)			2.77	(2.65-2.88)		
**Autopsy**					**2,831**	**< 0.001**			**343**	**< 0.001**
No	130,733 (14.3)	911,192	1 (ref.				1 (ref.)			
Non-forensic	46,862 (6.7)	46,862	0.43	(0.41-0.44)			0.72	(0.69-0.74)		
Forensic	42,074 (16.5)	42,074	1.18	(1.15-1.21)			1.06	(1.03-1.09)		

Logistic regression model, data from Norwegian Cause of Death Registry, 1996-2019
LR stat = Likelihood ratio statistic (-2LogL)

Table S1b – Logistic regression, factors correlated with minor garbage codes

Explanatory variable	Minor GC (%) N = 133,044	All deaths N = 1,000,128	Single predictor models				Multiple predictor model			
			OR	95 % CI	LR stat*	p	OR	95 % CI	LR stat*	p
**Year of death**					**4,202**	**< 0.001**			**6,862**	**< 0.001**
1996-1999	31,956 (18.0)	177,043	1 (ref.)				1 (ref.)			
2000-2004	35,683 (16.6)	214,402	0.91	(0.89-0.91)			0.87	(0.85-0.88)		
2005-2009	31,945 (15.6)	205,178	0.84	(0.82-0.85)			0.77	(0.75-0.78)		
2010-2014	26,939 (13.2)	203,647	0.69	(0.68-0.70)			0.60	(0.59-0.62)		
2015-2019	23,142 (11.6)	199,858	0.59	(0.58-0.61)			0.51	(0.50-0.52)		
**Sex**					**2,462**	**< 0.001**			**55**	**< 0.001**
Female	85,726 (16.7)	513,851	1 (ref.)				1 (ref.)			
Male	63,939 (13.1)	486,277	0.76	(0.75-0.76)			0.96	(0.95-0.97)		
**Age at death**					**21,869**	**< 0.001**			**8,424**	**< 0.001**
Under 1	81 (1.9)	4,264	1 (ref.)				1 (ref.)			
1-4	61 (5.9)	1,029	3.25	(2.31-4.56)			3.35	(2.37-4.70)		
5-14	72 (5.0)	1,453	2.69	(1.95-3.72)			2.66	(1.92-3.67)		
15-49	2337 (5.0)	46,910	2.71	(2.18-3.41)			2.90	(2.34-3.66)		
50-79	39,206 (10.2)	385,304	5.85	(4.73-7.35)			4.41	(3.57-5.55)		
80-89	66,844 (18.1)	369,224	11.4	(9.23-14.3)			7.16	(5.78-9.00)		
90-115	41,064 (21.4)	191,944	14.1	(11.4-17.7)			8.40	(6.79-10.6)		
**Place of death**					**19,123**	**< 0.001**			**6,820**	**< 0.001**
Hospital	41,135 (11.2)	366,855	1 (ref.)				1 (ref.)			
Nursing home	88,994 (20.5)	434,271	2.04	(2.02-2.07)			1.65	(1.63-1.68)		
At home	15,693 (10.5)	149,203	0.93	(0.91-0.95)			0.99	(0.97-1.01)		
Other known	2241 (6.5)	34,620	0.55	(0.52-0,57)			0.79	(0.75-0.83)		
Unknown	1602 (10.6)	15,179	0.93	(0.89-8.98)			1.12	(1.06-1.18)		
**Autopsy**					**9,958**	**< 0.001**			**2,097**	**< 0.001**
No	145,299 (15.9)	911,192	1 (ref.)				1 (ref.)			
Non-forensic	2634 (5.6)	46,862	0.31	(0.30-0.33)			0.49	(0.47-0.51)		
Forensic	1732 (4.1)	42,074	0.23	(0.22-0.24)			0.51	(0.48-0.54)		

Logistic regression model, data from Norwegian Cause of Death Registry, 1996-2019
LR stat = Likelihood ratio statistic (-2LogL)

Table S2a – The most prevalent garbage codes by sex

The most prevalent garbage codes in Norway 1996-2019			
Diagnostic code	*N*	*% of all deaths in group*	*% of GC in group*
**BY SEX**			
Females, N = 513,851			
**Major GC**	*80,245*	*15.6*	
I50 Heart failure	22,694	4.4	28.3
R54 Senility	7,871	1.5	9.8
R96 Sudden death	7,510	1.5	9.4
X59 Exposure to unspecified factor	5,719	1.1	7.1
A41 Other sepsis	3,359	0.7	4.2
**Minor GC**	*85,726*	*16.7*	
I64 Unspecified stroke	27,835	5.4	32.5
J18 Unspecified pneumonia	23,930	4.7	27.9
C80 Malignant neoplasm, unknown primary site	6,062	1.2	7.1
I69 Sequelae of cerebrovascular disease	5,533	1.1	6.5
E14 Unspecified diabetes mellitus	5,471	1.1	6.4
Males, N = 486,277			
**Major GC**	*60,559*	*12.5*	
I50 Heart failure	13,989	2.9	23.1
R96 Sudden death	6,617	1.4	10.9
X59 Exposure to unspecified factor	3,696	0.8	6.1
R99 Unknown cause of death	3,282	0.7	5.4
A41 Other sepsis	3,215	0.7	5.3
**Minor GC**	*63,939*	*13.1*	
J18 Unspecified pneumonia	17,823	3.7	27.9
I64 Unspecified stroke	15,979	3.3	25.0
E14 Unspecified diabetes mellitus	4,954	1.0	7.7
C80 Malignant neoplasm, unknown primary site	4,951	1.0	7.7
I69 Sequelae of cerebrovascular disease	4,591	0.9	7.2

Data source: NCoDR

Table S2b – The most prevalent garbage codes by age group

The most prevalent garbage codes in Norway 1996-2019

Diagnostic code	*N*	*% of all deaths in group*	*% of GC in group*
**BY AGE AT DEATH**			
Under 1 year, N = 4,264			
**Major GC**	*46*	*1.1*	
R99 Unknown cause of death	16	0.4	34.8
A41 Other sepsis	8	0.2	17.4
A40 Streptococcal sepsis	4	0.1	8.7
W76 Other accidental hanging and strangulation	4	0.1	8.7
B34 Viral infection, unspecified	2	0.05	4.3
G93 Unspecified disorder of brain	2	0.05	4.3
**Minor GC**	*81*	*1.9*	
J18 Unspecified pneumonia	13	0.3	16.0
Q89 Other congenital malformations	12	0.3	14.8
P23 Congenital pneumonia	8	0.2	9.9
D82 Immunodeficiency with other major defects	6	0.1	7.4
Q99 Other chromosomal abnormalities	6	0.1	7.4
1-4 years, N = 1,029			
**Major GC**	*89*	*8.6*	
G80 Cerebral palsy	13	1.3	14.6
R99 Unknown cause of death	12	1.2	13.5
W76 Other accidental hanging and strangulation	11	1.1	12.4
A40 Streptococcal sepsis	10	1.0	11.2
A41 Other sepsis	9	0.9	10.1
**Minor GC**	*61*	*5.9*	
J18 Unspecified pneumonia	8	0.8	13.1
C74 Malignant neoplasm of adrenal gland	6	0.6	9.8
G03 Meningitis, unspecified	5	0.5	8.2
V89 Unspecified traffic accident	4	0.4	6.6
C76 Malignant neoplasm, ill-defined site	3	0.3	4.9

D81 Combined immunodeficiency, unspecified	3	0.3	4.9
G00 Bacterial meningitis, unspecified	3	0.3	4.9
J22 Unspecified lower respiratory infection	3	0.3	4.9
Q89 Other congenital malformations	3	0.3	4.9
5-14 years, N = 1,453			
**Major GC**	*142*	*9.8*	
G80 Cerebral palsy	61	4.2	43.0
R99 Unknown cause of death	14	1.0	9.9
W76 Other accidental hanging and strangulation	14	1.0	9.9
F84 Pervasive developmental disorders	8	0.6	5.6
G93 Unspecified disorder of brain	7	0.5	4.9
**Minor GC**	*72*	*5.0*	
C74 Malignant neoplasm of adrenal gland	15	1.0	20.8
I42 Cardiomyopathy, unspecified	10	0.7	13.9
I49 Cardiac arrhythmia, unspecified	6	0.4	8.3
C91 Lymphoid leukemia, unspecified	5	0.3	6.9
I45 Other cardiac conduction disorders	5	0.3	6.9
V89 Unspecified traffic accident	5	0.3	6.9
15-49 years, N = 46,910			
**Major GC**	*6,848*	*14.6*	
X42 Accidental poisoning by narcotics and psychodysleptics	2,352	5.0	34.3
X44 Accidental poisoning by unspecified drugs	799	1.7	11.7
R99 Unknown cause of death	608	1.3	8.9
F19 Mental and behavioural disorders due to multiple drug use	552	1.2	8.1
X41 Accidental poisoning by sedatives	496	1.1	7.2
**Minor GC**	*2,337*	*5.0*	
C80 Malignant neoplasm, unknown primary site	289	0.6	12.4
E14 Unspecified diabetes mellitus	273	0.6	11.7
V89 Unspecified traffic accident	224	0.5	9.6

I51 Ill-defined heart disease	182	0.4	7.8
X84 Intentional self-harm by unspecified means	167	0.4	7.1
50-79 years, N = 385,304			
**Major GC**	*34,114*	*8.9*	
I50 Heart failure	5,675	1.5	16.6
R96 Sudden death	4,863	1.3	14.3
R99 Unknown cause of death	2,659	0.7	7.8
I10 Essential hypertension	2,112	0.5	6.2
A41 Other sepsis	2,056	0.5	6.0
**Minor GC**	*39,206*	*10.2*	
I64 Unspecified stroke	9,590	2.5	24.5
J18 Unspecified pneumonia	5,856	1.5	14.9
C80 Malignant neoplasm, unknown primary site	5,333	1.4	13.6
E14 Unspecified diabetes mellitus	3,901	1.0	10.0
I51 Ill-defined heart disease	2,559	0.5	6.5
80-89 years, N = 369,224			
**Major GC**	*55,294*	*15.0*	
I50 Heart failure	16,751	4.5	30.3
R96 Sudden death	5,366	1.5	9.7
X59 Exposure to unspecified factor	4,333	1.2	7.8
R54 Senility	3,332	0.9	6.0
N19 Unspecified kidney failure	3,075	0.8	5.6
**Minor GC**	*66,844*	*18.1*	
I64 Unspecified stroke	22,216	6.0	33.2
J18 Unspecified pneumonia	19,208	5.2	28.7
I69 Sequelae of cerebrovascular disease	5,095	1.4	7.6
E14 Unspecified diabetes mellitus	4,338	1.2	6.5
C80 Malignant neoplasm, unknown primary site	4,057	1.1	6.1
90 years and above, N = 191,944			
**Major GC**	*44,271*	*23.1*	

I50 Heart failure	14,183	7.4	32.0
R54 Senility	6,660	3.5	15.0
X59 Exposure to unspecified factor	3,628	1.9	8.2
R96 Sudden death	3,540	1.8	8.0
N19 Unspecified kidney failure	1,912	1.0	4.2
**Minor GC**	*41,064*	*21.4*	
J18 Unspecified pneumonia	16,510	8.6	40.2
I64 Unspecified stroke	11,963	6.2	29.1
I69 Sequelae of cerebrovascular disease	2,544	1.3	6.2
I51 Ill-defined heart disease	2,350	1.2	5.7
E14 Unspecified diabetes mellitus	1,912	1.0	4.7

Data source: NCoDR

Table S2c – The most prevalent garbage codes by place of death

The most prevalent garbage codes in Norway 1996-2019

Diagnostic code	*N*	*% of all deaths in group*	*% of GC in group*
**BY PLACE OF DEATH**			
Hospital, N = 366,855			
**Major GC**	*33,531*	*9.1*	
I50 Heart failure	7,837	2.1	23.4
A41 Other sepsis	4,750	1.3	14.2
X59 Exposure to unspecified factor	3,441	0.9	10.3
N19 Unspecified kidney failure	2,058	0.6	6.1
I26 Pulmonary embolism	1,785	0.5	5.3
**Minor GC**	*41,135*	*11.2*	
J18 Unspecified pneumonia	11,819	3.2	28.7
I64 Unspecified stroke	10,971	3.0	26.7
C80 Malignant neoplasm, unknown primary site	5,091	1.4	12.4
E14 Unspecified diabetes mellitus	2,054	0.6	5.0
I69 Sequelae of cerebrovascular disease	1,132	0.3	2.8
Nursing home, N = 434,271			
**Major GC**	*68,638*	*15.8*	
I50 Heart failure	23,301	5.4	33.9
R54 Senility	8,923	2.1	13.0
X59 Exposure to unspecified factor	5,236	1.2	7.6
R96 Sudden death	4,742	1.1	6.9
N19 Unspecified kidney failure	3,509	0.8	5.1
**Minor GC**	*88,994*	*20.5*	
I64 Unspecified stroke	28,921	6.7	32.5
J18 Unspecified pneumonia	27,211	6.3	30.6
I69 Sequelae of cerebrovascular disease	7,963	1.8	8.9
E14 Unspecified diabetes mellitus	5,530	1.3	6.2
C80 Malignant neoplasm, unknown primary site	4,637	1.1	5.2

At home, N = 149,203			
**Major GC**	*28,545*	*19.1*	
R96 Sudden death	7,140	4.8	25.0
I50 Heart failure	4,671	3.1	16.4
I10 Essential hypertension	2,556	1.7	9.0
R99 Unknown cause of death	2,138	1.4	7.5
I46 Cardiac arrest	1,439	1.0	5.0
**Minor GC**	*15,693*	*10.5*	
I64 Unspecified stroke	3,231	2.2	20.6
I51 Ill-defined heart disease	2,677	1.8	17.1
E14 Unspecified diabetes mellitus	2,382	1.6	15.2
J18 Unspecified pneumonia	2,142	1.4	13.6
C80 Malignant neoplasm, unknown primary site	1,115	0.7	7.1
Other known place, N = 34,620			
**Major GC**	6,796	19.6	
R99 Unknown cause of death	1,817	5.2	26.7
R96 Sudden death	1,319	3.8	19.4
X42 Accidental poisoning by narcotics and psychodysleptics	766	2.2	11.3
I46 Cardiac arrest	471	1.4	6.9
I50 Heart failure	432	1.2	6.4
**Minor GC**	2,241	6.5	
I51 Ill-defined heart disease	432	1.2	19.3
I64 Unspecified stroke	348	1.0	15.5
E14 Unspecified diabetes mellitus	264	0.8	11.8
V89 Unspecified traffic accident	251	0.7	11.2
J18 Unspecified pneumonia	223	0.6	10.0
Unknown place of death, N = 15,179			
**Major GC**	3,294	21.7	
R99 Unknown cause of death	626	4.1	19.0
R96 Sudden death	466	3.1	14.1

I50 Heart failure	442	2.9	13.4
X42 Accidental poisoning by narcotics and psychodysleptics	418	2.8	12.7
R54 Senility	151	1.0	4.6
**Minor GC**	1,602	10.6	
J18 Unspecified pneumonia	358	2.4	22.3
I64 Unspecified stroke	343	2.3	21.4
E14 Unspecified diabetes mellitus	195	1.3	12.2
I51 Ill-defined heart disease	189	1.2	11.8
I69 Sequelae of cerebrovascular disease	100	0.7	6.2

Data source: NCoDR

Table S2d - The most prevalent garbage codes by autopsy type

The most prevalent garbage codes in Norway 1996-2019			
Diagnostic code	*N*	*% of all deaths in group*	*% of GC in group*
**BY AUTOPSY TYPE**			
No autopsy, N = 911,192			
**Major GC**	*130,733*	*14.3*	
I50 Heart failure	36,496	4.0	27.9
R96 Sudden death	13,904	1.5	10.6
R54 Senility	10,297	1.1	7.9
X59 Exposure to unspecified factor	9,011	1.0	6.0
A41 Other sepsis	6,280	0.7	4.8
**Minor GC**	*145,299*	*15.9*	
I64 Unspecified stroke	43,722	4.8	30.1
J18 Unspecified pneumonia	40,614	4.5	28.0
C80 Malignant neoplasm, unknown primary site	10,723	1.2	7.4
I69 Sequelae of cerebrovascular disease	10,087	1.1	6.9
E14 Unspecified diabetes mellitus	10,058	1.1	6.9
Non-forensic autopsy, N = 46,862			
**Major GC**	*3,118*	*6.7*	
I26 Pulmonary embolism	493	1.1	15.8
A41 Other sepsis	256	0.5	8.2
X59 Exposure to unspecified factor	221	0.5	7.1
E85 Amyloidosis	204	0.4	6.5
J85 Abscess of lung and mediastinum	122	0.3	3.9
**Minor GC**	*2,634*	*5.6*	
J18 Unspecified pneumonia	780	1.7	29.6
C80 Malignant neoplasm, unknown primary site	274	0.6	10.4
I51 Ill-defined heart disease	252	0.5	9.6
I42 Cardiomyopathy (unspecified)	169	0.4	6.4
E14 Unspecified diabetes mellitus	133	0.3	5.0

Forensic autopsy, N = 42,074			
**Major GC**	*6,953*	*16.5*	
X42 Accidental poisoning by narcotics and psychodysleptics	2,639	6.3	38.0
X44 Accidental poisoning by unspecified drugs	947	2.3	13.6
X41 Accidental poisoning by sedatives	746	1.8	10.7
R99 Unknown cause of death	570	1.4	8.2
F19 Mental and behavioural disorders due to multiple drug use	356	0.8	5.1
**Minor GC**	*1,732*	*4.1*	
I51 Ill-defined heart disease	504	1.2	29.1
J18 Unspecified pneumonia	359	0.9	20.7
E14 Unspecified diabetes mellitus	234	0.6	13.5
I42 Cardiomyopathy (unspecified)	111	0.3	6.4
V89 Unspecified traffic accident	78	0.2	4.5

Data source: NCoDR

Table S3a - The most prevalent non-garbage codes by major garbage code

Non-garbage codes according to GBD Cause List, detail level 3

(Note that there may be more than one non-garbage code in each death.)

Diagnostic code	*N*	% of all deaths in group
**All Major GC, N = 140,804**	*46,287*	*32.9*
Alzheimer disease and other dementias	10,348	7.4
Ischaemic heart disease	6,563	4.7
Atrial fibrillation and flutter	5,183	3.6
Chronic obstructive pulmonary disease	4,866	3.4
Effects of medical treatment	4,186	2.9
**I50 Heart failure, N = 36,683**	*12,844*	*35.0*
Alzheimer disease and other dementias	3,342	9.1
Chronic obstructive pulmonary disease	1,869	5.1
Atrial fibrillation and flutter	1,520	4.1
Urinary diseases	765	2.1
Cerebrovascular diseases	548	1.5
**R96 Sudden death, N = 14,127**	*328*	*2.3*
Prostate cancer	54	0.4
Colorectal cancer	48	0.3
Skin cancer	44	0.3
Bladder cancer	35	0.3
Breast cancer	28	0.3
**R54 Senility, N = 10,298**	*279*	*2.7*
Skin cancer	64	0.6
Breast cancer	46	0.4
Prostate cancer	29	0.3
Colorectal cancer	26	0.3
Bladder cancer	16	0.2
**X59 Exposure to unspecified factor, N = 9,415**	*6,442*	*68.4*
Effects of medical treatment	2,543	27.0

Alzheimer disease and other dementias	1,966	20.9
Ischaemic heart disease	1,274	13.5
Atrial fibrillation and flutter	774	8.2
Chronic obstructive pulmonary disease	492	5.2
**A41 Other sepsis, N = 6,574**	*3,444*	*52.3*
Ischaemic heart disease	919	14.0
Alzheimer disease and other dementias	564	8.6
Atrial fibrillation and flutter	353	5.4
Chronic obstructive pulmonary disease	328	5.0
Urinary diseases	221	3.4

Table S3b - The most prevalent non-garbage codes by minor garbage code

Non-garbage codes according to GBD Cause List, detail level 3
(Note that there may be more than one non-garbage code in each death.)

Diagnostic code	*N*	% of all deaths in group
**All Minor GC, N = 149,665**	*58,393*	*39.0*
Alzheimer disease and other dementias	14,920	10.0
Ischaemic heart disease	12,040	8.0
Atrial fibrillation and flutter	6,505	4.4
Urinary diseases	3,728	2.5
Chronic obstructive pulmonary disease	3,621	2.4
**I64 Unspecified stroke, N = 43,814**	*18,156*	*41.4*
Alzheimer disease and other dementias	6,204	14.2
Ischaemic heart disease	2,972	6.8
Atrial fibrillation and flutter	2,287	5.2
Cerebrovascular diseases	1,138	2.6
Chronic obstructive pulmonary disease	1,108	2.5
**J18 Unspecified pneumonia, N = 41,753**	*12,226*	*29.3*
Alzheimer disease and other dementias	3,249	7.8
Ischaemic heart disease	2,992	7.2
Atrial fibrillation and flutter	1,471	3.5
Urinary diseases	666	2.6
Diabetes mellitus	577	1.4
**C80 Malignant neoplasm, site unknown, N = 11,013**	*2,956*	*26.8*
Ischaemic heart disease	549	5.0
Chronic obstructive pulmonary disease	432	3.9
Alzheimer disease and other dementias	406	3.7
Atrial fibrillation and flutter	267	2.4
Endocrine, metabolic, blood, and immune disorders	139	1.3
**E14 Unspecified diabetes mellitus, N = 10,425**	*6189*	*59.4*
Ischaemic heart disease	3,045	29.2

Alzheimer disease and other dementias	1,050	10.1
Atrial fibrillation and flutter	623	6.0
Chronic kidney disease	501	4.8
Cerebrovascular diseases	453	4.4
**I69 Sequelae of cerebrovascular disease, N = 10,124**	*4,538*	*44.8*
Alzheimer disease and other dementias	1,267	12.5
Ischaemic heart disease	728	7.2
Urinary diseases	511	5.1
Atrial fibrillation and flutter	503	5.0
Cerebrovascular diseases	235	2.3

Table S4 - Definition of garbage codes

The definition is copied from Table S5 in the methods appendix to GBD 2019 Diseases and Injuries Collaborators. GBD 2019: Global burden of 369 diseases and injuries in 204 countries and territories, 1990–2019: a systematic analysis for the Global Burden of Disease Study 2019. Lancet. 2020;396(10258):1204-22. (Corrected for some obvious typographic errors.)

**Level 1 (very high)**	Codes with serious policy implications	The true UCD might belong to any of the three broad groups of causes of death (communicable, maternal, neonatal and nutritional disease; non-communicable diseases; injuries).<br>E.g. sepsis
**Level 2 (high)**	Codes with substantial policy implications	The true UCD might belong to one (or at most two) of the three broad groups of causes of death.<br>E.g. unspecified injury
**Level 3 (medium)**	Codes with important policy implications	The true UCD is likely to be within the same ICD chapter.<br>E.g. cancer of unknown site
**Level 4 (low)**	Codes with limited policy implications	The true UCD is likely to be within a single disease or injury category.<br>E.g. unspecified stroke

Level 1 and 2 are major garbage codes, level 3 and 4 minor.

**Garbage Code (GBD Level 1)**
A40-A41.9, A48.0, A48.3, A49.0-A49.1, A59-A59.9, A71-A71.9, A74.0,<br>B07-B07.9, B30-B30.9, B35-B36.9, B85-B85.4, B87-B88.9, B94.0,<br>D50-D50.0, D50.9, D62-D63.0, D63.8-D64, D64.1-D65.9, D68, D69.9,<br>E15, E16, E50-E50.9, E64.1, E85.3-E87.6, E87.8-E87.9,<br>F06.2-F06.4, F07.2, F09-F09.9, F19-F23.9, F25-F49, F51-F99.0,<br>G06-G08.0, G32-G32.8, G43-G44.2, G44.4-G44.8, G47-G47.2, G47.4-G47.9, G50-G60.9, G62-G62.0, G62.2-G65.2, G80-G83.9, G89-G89.4, G91-G91.2, G91.4-G93, G93.1-G93.2, G93.4-G93.6, G94.0-G94.8, G99-G99.8,<br>H00-H05, H05.2-H69.9, H71-H99,<br>I26-I26.9, I31.2-I31.4, I46-I46.9, I50.0-I50.4, I76, I95-I95.1, I95.8-I95.9,

J69-J69.9, J80-J80.9, J81.0, J85-J85.3, J86-J86.9, J93-J93.1, J93.8-J93.9, J94.2, J96-J96.9, J98.1-J98.3,<br>K00-K19, K30, K65-K66.1, K66.9, K68.1-K68.9, K71-K71.6, K71.8-K72.9, K75.0,<br>L20-L30.9, L40-L50.9, L52-L54.8, L56-L56.2, L56.4-L56.5, L57-L57.9, L59-L68.9, L70-L76.8, L80-L87.9, L90-L92.9, L94-L96, L98.5-L99.8,<br>M04, M10-M12.0, M12.2-M29, M37-M39, M43.2-M49, M49.2-M64, M65.1-M71, M71.2-M72.4, M72.8-M73, M73.8-M79.9, M83-M86.2, M86.5-M86.9, M87.2-M87.9, M89.1-M89.4, M90-M99.9,<br>N17-N17.9, N19-N19.9, N32.1-N32.2, N32.8-N33.8, N35-N35.9, N37-N37.8, N39.3-N39.8, N42-N43.4, N44.1-N44.8, N46-N48.9, N50-N53.9, N61-N64.9, N82-N82.9, N91-N91.5, N95, N95.1-N95.9, N97-N97.9,<br>R02-R02.9, R03.1, R07.0, R08-R09, R09.3, R11-R12.0, R14-R19.6, R19.8-R23, R23.1-R30.9, R32-R50.1, R50.8-R57.9, R58.0-R72.9, R74-R78, R78.6-R94.8, R96-R99.9,<br>U05, U07-U81, U89.9-U99,<br>X40-X44.9, X46-X46.9, X49-X49.9,<br>Y10-Y14.9, Y16-Y19.9,<br>Z00-Z15.8, Z17-unsp.
**Garbage Code (GBD Level 2)**
A14.9, A29-A30.9, A45-A45.9, A47-A48, A48.8-A49, A49.3-A49.9, A61-A62, A72-A73, A76, A97,<br>B08-B09, B11-B14, B28-B29, B31-B32.4, B34-B34.9, B61-B62, B68-B68.9, B73-B74.2, B76-B76.9, B78-B81.8, B84, B92-B94, B94.8-B94.9, B95.6, B97.3, B97.7-B99.9,<br>D59, D59.4, D59.8-D59.9,<br>F17-F17.9,<br>G44.3, G91.3, G93.0, G93.3,<br>I10-I10.9, I15-I15.9, I27, I27.8-I27.9, I50, I50.8-I50.9, I67.4, I70-I70.1, I70.9, I74-I75.8,<br>J81, J81.1, J90-J90.0, J94-J94.1, J94.8-J94.9,<br>K92.0-K92.2,<br>N70-N71.9, N73-N74.0, N74.2-N74.8,<br>R03-R03.0, R04-R06.9, R09.0-R09.2, R09.8-R10.9, R13-R13.9, R23.0, R58,<br>S00-T98.3,<br>W47-W48, W63, W71-W72, W76-W76.9, W82, W95-W97, W98,<br>X07, X55-X56, X59-X59.9,<br>Y20-Y34.9, Y86-Y87, Y87.2, Y89, Y89.9-Y99.9

**Garbage Code (GBD Level 3)**
A01, A31-A31.9, A42-A44.9, A49.2, A64-A64.0, A99-A99.0,<br>B17, B17.1, B17.8-B17.9, B19-B19.0, B19.2-B19.9, B37-B46.9, B49-B49.9, B55, B55.1-B55.9, B58-B59.9, B89, B94.2,<br>C14-C14.9, C22.9, C26-C29, C35-C36, C39-C39.9, C42, C46-C46.9, C55-C55.9, C57.9, C59, C63.9, C68, C68.9, C74-C74.9, C75.9-C80.9, C87, C97,<br>D00.0, D01, D01.4-D02, D02.4-D02.9, D07, D07.3, D07.6-D09, D09.1, D09.7, D09.9-D10, D10.9, D13, D13.9-D14, D14.4, D17-D21.9, D28, D28.9-D29, D29.9-D30, D30.9, D36.0, D36.9-D37.0, D37.6-D38, D38.6-D39.0, D39.7, D39.9-D40, D40.9-D41, D41.9, D44, D44.9, D48, D48.7-D49.1, D49.5, D49.7-D49.9, D54, D75.9, D79-D85, D87-D88, D89.8-D99,<br>E07.8-E08.9, E17-E19, E34.0, E34.9-E35.8, E37-E39, E47-E49, E62, E69, E87.7, E90-E998,<br>F04-F06.1, F06.5-F07.0, F07.8-F08, F50, F50.8-F50.9, G09-G09.9, G15-G19, G21, G21.2, G21.4-G22.0, G27-G29, G33-G34, G38-G39, G42, G48-G49, G66-G69, G74-G79, G84-G88, G93.8-G94, G96-G96.9, G98-G98.9,<br>I00.0, I03-I04, I14-I14, I16-I19, I29-I29.9, I44-I45.9, I49-I49.9, I51, I51.6-I59, I90-I94, I96-I96.9, I98.4-I98.8, I99-ID5.9, J02.9, J03.9, J04.3, J06, J06.9, J40-J40.9, J47-J59, J71-J79, J81.9, J83, J85.9, J87-J89, J90.9, J93.6, J97-J98.0, J98.4-J99.8,<br>K21-K21.9, K22.7, K31.9-K34, K39, K47-K49, K53-K54, K63-K63.4, K63.8-K63.9, K69, K70.4-K70.9, K78-K79, K84, K87-K89, K92, K92.9-K93, K96-K99,<br>L06-L07, L09, L15-L19, L31-L39, L69, L77-L79,<br>N09, N13-N13.5, N13.7-N13.9, N24, N28.8-N28.9, N38, N39.9-N40.9, N54-N59, N66-N69, N78-N79, N84, N84.2-N86, N88-N90.9, N92-N94.9, N95.0,<br>O08-O08.9, O17-O19, O27, O37-O39, O49-O59, O78-O79, O93-O95.9,<br>P06, P16-P18, P30-P34.2, P40-P49, P62-P69, P73, P79, P82, P85-P89, P96.9-P99.9,<br>Q08-Q10.3, Q19, Q29-Q29, Q36.0-Q36.9, Q46-Q49, Q88, Q89.9, Q94, Q99.9<br>R00-R01.2, R07, R07.1-R07.9, R31-R31.9
**Garbage Code (GBD Level 4)**
B16.9, B64, B82-B82.9, B83.9,<br>C69, C69.9, C91.1, C91.4-C91.5, C91.7-C91.9, C92.7-C92.9, C93.2, C93.5-C93.7, C93.9,<br>E12-E14.9,<br>G00, G00.9-G02.8, G03.9,<br>I37.9, I42-I42.0, I42.9, I51.5, I64-I64.9, I67, I67.8-I68, I68.8-I69, I69.4-I69.9,

J07-J08, J15.9, J17-J19.6, J22-J29, J64-J64.9,<br>P23, P23.5-P23.9, P37.3-P37.4,<br>R73-R73.9,<br>V87-V87.1, V87.4-V88.1, V88.4-V89.9, V99-V99.0,<br>X84-X84.9, Y09-Y09.9, Y85-Y85.9
